# Should ultrasound guided percutaneous renal biopsy in children be done in a day care setting?

**DOI:** 10.4103/0971-4065.62092

**Published:** 2010

**Authors:** V. Mahajan, D. Suri, A. Saxena, R. Nada

**Affiliations:** Department of Pediatrics, PGIMER, Chandigarh, India; 1 Department of Radiology, PGIMER, Chandigarh, India; 2 Department of Histopathology, PGIMER, Chandigarh, India

**Keywords:** Complications, daycare, percutaneous renal biopsy

## Abstract

Percutaneous renal biopsy (PRB) is an important diagnostic tool in pediatric nephrology units. But controversy exists whether the procedure can be done in the day care setting. This study was done to document complications of PRB done with automated gun under continuous ultrasonographic guidance and to find whether the procedure can be undertaken as a day care procedure. Retrospective analysis of 67 PRBs is presented. A total of 44% (*n = *30) minor and 12% (*n = *8) major complications such as gross hematuria, perinephric hematoma, and hemodynamic instability were observed through the study period. All major and 90% of minor complications were detected within four hours in the current study. The procedure may be undertaken in the day care setting with strict pre and postprocedure monitoring up to eight hours in children with normal blood pressures, renal functions, hemoglobin concentrations, and coagulation parameters.

## Introduction

Evaluation of renal histology is essential for the proper diagnosis and prognostication of various renal parenchymal diseases. Percutaneous renal biopsy (PRB) has become indispensible tool in obtaining renal tissue for histological evaluation. The success of the procedure is defined not only by the ability to obtain adequate tissue for diagnosis but equally, if not more importantly, by the safety profile. Early pediatric RB series describe the use of various manually operated cutting needles with static-image ultrasound being used for localization of the kidney.[1] The use of the biopsy gun for pediatric RB was first introduced in 1989.[2] Safety of the procedure has been well established in the literature. The standard of care after RB has included bed rest with close observation for 24 hours.[3] However, because of the current safety profile of the procedure and in efforts to reduce the cost of care, it has been performed as a “day care procedure,” without any increase in complications.[4,5] However, some prefer overnight observation in the hospital.[6] The current study was carried out to document complications of PRB and explore the possibility of doing procedure in day care setting, using a retrospective analysis of renal biopsies performed in the nephrology unit, done with the automated spring loaded biopsy gun with the aim of finding out whether PRB can be undertaken as a day care procedure in children.

## Materials and Methods

Present study was carried out in Pediatric Nephrology unit of a tertiary care teaching hospital of northern India. A total of 67 renal biopsies done from Nov 01, 2006 to Oct 31, 2007 were retrospectively analyzed.

### Renal biopsy protocol

All biopsies were performed as an indoor procedure as a unit policy. A written informed consent was obtained prior to the procedure from the parents/legal guardians. A coagulation profile, hemogram and blood group testing were done prior to the biopsy. Abnormal homeostasis, uncontrolled hypertension and active urinary tract infection was excluded in all patients before RB. The biopsies were done by the doctors skilled in both ultrasonography (USG) and RB technique under ultrasonic guidance in the presence of trained personnel skilled in pediatric advanced life support. Children were closely monitored clinically during and after the procedure. All RB were done with patients lying in prone position. The children were sedated using midazolam 0.1 mg/kg. Ketamine 1 mg/kg was used in addition where required. The lower pole of the left kidney was used for puncture in native kidneys. Bard gun max.4 disposable biopsy instrument (Bard Limited UK), size 18G*16 cm length was used. The renal tissue obtained was sent for (i) light-microscopy, (ii) immunofluorescence and (iii) electron-microscopy.

### Monitoring

After the procedure, patients were advised to rest in bed for 24 hours and were clinically monitored periodically for any deterioration. An immediate postbiopsy ultrasound was done in all children to detect perinephric hematoma. Repeat ultrasounds were done in all children who developed gross hematuria. Serial urine microscopic examination s were done to look for hematuria. Inadequacy of biopsy tissue was defined as inability to get renal tissue or obtaining renal tissue containing less than 5 glomeruli, making it insufficient for diagnosis.[7] Minor complications included microscopic hematuria. Major complications included gross hematuria, perinephric hematoma, requirement of blood transfusion, biopsy site infection, hypertension, arteriovenous fistula, organ loss, hemodynamic instability and death.

Descriptive statistics were used to define baseline variables. Statistical analysis was performed using the independent ‘*t*’ test for continuous data and Chi-square or Fisher's exact test for categorical data. Multivariate analysis using logistic regression was performed to predict independent baseline factors for the major complications after RB. The baseline variables examined included age, blood pressure, serum creatinine, hemoglobin concentration and prothrombin time index. *P* value of < 0.05 was considered significant.

## Results

A total of 67 consecutive biopsies performed on 65 children (51 boys and 14 girls), were analyzed. All biopsies were performed on native kidney. Nephrotic syndrome was the most frequent indication in 42 (64.6%) cases, out of which 23 (54%) were steroid resistant nephrotic syndrome. The other indications of RB are as summarized in Table 1. There were 11 cases of acute renal failure and their range of serum creatinine was 1.3–15.7 mg/dl. There were failed biopsies in 3 children (4%). Among 64 diagnostic samples, the most frequent histology was focal segmental glomerulosclerosis in 16 (25%) of the samples.

**Table 1:** Indications for renal biopsy (n = 65)

Indication	No. of patient (%)
Nephrotic syndrome	
Steroid resistant nephrotic syndrome	23 (35.4)
Frequent relapsing nephrotic syndrome	16 (24.6)
Nephrotic-nephritic syndrome	3 (4.6)
	
Rapidly progressive renal failure	
Rapidly progressive glomerulonephritis	6 (9.1)
Acute tubular necrosis	1 (1.5)
D(−) Hemolytic uremic syndrome	4 (6.1)
D(+) Hemolytic uremic syndrome	1(1.5)
	
Systemic diseases	
Lupus nephritis	7 (10.7)
Henoch scholein purpura	3 (4.6)
	
Hereditary disorders	
Alport's syndrome	1 (1.5)

Postbiopsy complications are as shown in Table 2. In 51.8% patients, there were no complications. Minor complications like microscopic hematuria occurred in 30 (44%) cases. Sixtyfive percent of minor complications were detected within 4 hours of procedure, 90% within 8 hours and 100% in first 24 hours. A total of 8 (12%) major complications were recorded during the study period. Gross hematuria was present in 3 (4%) cases, which lasted for <48 hours. Perinephric hematoma was identified in 5 (7%) children. Two children (3%) developed life threatening complications like hemodynamic compromise, one of whom required blood transfusion due to severe hematuria and hematoma formation. Both patients were resuscitated immediately with volume expansion and fluids. Urinary tract infection (postprocedure) was documented in only one child. No organ loss or death was reported. Pain was not a significant complaint in any of the patients, with a very few of them requiring paracetamol. All major complications were detected with in first 4 hours of procedure. Table 3 shows the timings of detection of major complications.

**Table 2:** Frequency of complications following the renal biopsy and comparison between the various studies

Complications	Al Rashid *et al*. (N = 120) 1990[10](%)	Chesney *et al*. (N = 75) 1996[12](%)	Nammalwar *et al*. (N = 250) 2006[7](%)	Current study (N = 67) 2007(%)
Minor complications				
Microscopic hematuria	70	——	82 (32.8)	30 (44)
				
Major complications
Gross hematuria	32 (26.7)	4 (5.3)	42 (16.8)	3 (4)
Perirenal hematoma	19 (15.1)	1 (1.2)	15 (6)	5 (7)
RB site infection	——	1 (1.2)	2 (0.8)	0
Failed biopsy	17 (14.5)	3 (4)	12 (4.8)	3 (4)
Hypertension	3 (2.5)	——	——	0
Arteriovenous fistula	2 (1.7)	——	——	0
Hemodynamic instability	——	——	——	2 (3)
Death	1 (0.8)	——	0	0
Organ loss	1 (1.5)	——	1 (0.4)	0
Blood transfusion	5 (5.2)	——	——	1 (1.5)

—— = Not described in the study

**Table 3:** Showing time of occurrence of major complications

Major complication	Time of occurrence (hours)	Primary diagnosis	Intervention
Hemodynamic instability	1½	RPGN	Inotropic support
Perinephric hematoma	3	SRNS	Hydration
Hemodynamic instability, gross hematuria, perinephric hematoma	2½	SRNS	Blood transfusion, fluid bolus, hydration
Gross hematuria	1½	FRNS	Hydration
Gross hematuria	2½	Familial nephrotic syndrome	Hydration
Perinephric hematoma	2	IGA nephropathy	Hydration
Perinephric hematoma	2	SRNS	Hydration
Perinephric hematoma	3	Atypical HUS	Hydration

Children with major complications were compared with those who did not have major complications. The two groups were balanced in terms of baseline demographic variables. There was no difference in serum creatinine, incidence of high blood pressure, hemoglobin concentration and PTI [Table 4]. After applying logistic regression, none of the factors emerged as independent predictor variable for major complications.

**Table 4:** Patient characteristics at biopsy

Characteristics	With major complications (n = 8)	Without major complications (n = 59)	*P*value
Age (years)	7.1±2.9	8.0±3.4	0.4
Males (%)	100	75	0.09
Serum creatinine (mg/dl)	1 (1,3.5)	1 (1,2)	0.98
Hemoglobin (gm%)	12.1 (3.3)	11.1±2.1	0.5
PTI	100 (100, 100)	100 (100, 100)	0.7

## Discussion

Present study shows 44% incidence of minor and 12% incidence of major complications. Ninety percent of minor and all major complications were detected with in first eight hours of biopsy. No factor could independently predict the occurrence of major complications.

RB contributes to diagnosis in 44–63% of cases, predicts treatment in 31–42% and aids prognosis in 32–57% of patients.[5,8] It was diagnostic in 95.5% of our patients which is comparable to world literature.[7,9] The minor complications in the form of microscopic hematuria is universal and has been reported in nearly 70–100% patients.[6,10] However, it has been observed in only 16% cases in a study from south India.[7] In our study, microscopic hematuria was seen in 30% patients. Postprocedural gross haematuria may develop in around 10% of patients (range 3–27%).[9] In current series, it occurred in only 3% children. Major complications in the form of hemodynamic compromise after biopsy occurred in 2 (3%) children which is comparable to previous studies.[11] Clinically significant perirenal hematoma occurs in 6% of cases.[9] In our study, it was found in 7% children. No infections occurred at the biopsy site in our study patients.

Children undergoing RB were associated with 2–3% life threatening complications which emphasizes the need for strict pre and postprocedure monitoring. Due to the low complication rate, the day care procedure has been suggested for RB.[4,5] The primary underlying reason is the cost saving. In one series outpatient biopsy costs USD1200 less than the inpatient procedure.[12] In our setting the day care procedure will cost approximately INR400 less than the inpatient procedure. Although all patients were admitted in the current series, but the data shows that PRB is feasible in the day care settings as all the major complications and 90% of minor complications occurred within 8 hours in the current series. In a study of 750 adult renal biopsies, 67% complications were identified by 8 hours, and 89% by 24 hours.[3] Similarly Marwah*etal*.[13] in series of 394 adult patients could identify 77% complications by 8 hours and 95% by 12 hours. There are chances of missing 20–30% complications in adult patients if observation was done till 8 hours only. We would have missed only 10% of microscopic hematuria, if these biopsies were done in day care settings.

There is always a concern regarding the generalization of the results of small series like ours. Although it seems convincing from the current series, doing RB as day care procedure should employ risk stratification. Children with high blood pressures, high creatinine values, requiring multiple passes to obtain tissue and borderline hemoglobin or coagulation parameters may be monitored for longer duration and inpatient biopsy.[3,13] In all other children an observation of 8 hours after RB may be sufficient.

## Conclusion

PRB may be undertaken as a day care procedure in children with normal blood pressures, renal functions, hemoglobin concentrations and coagulation parameters. Strict pre and postprocedure monitoring up to 8 hours is mandatory due to the possibility of the serious complications associated with PRB.
